# Benzene-1,3,5-tricarboxylic acid–1,2-bis­(1,2,4-triazol-4-yl)ethane–water (4/1/2)

**DOI:** 10.1107/S1600536808015808

**Published:** 2008-06-07

**Authors:** Hesham A. Habib, Christoph Janiak

**Affiliations:** a Institut für Anorganische und Analytische Chemie, Universität Freiburg, Albertstrasse 21, D-79104 Freiburg, Germany

## Abstract

The title compound, 4C_9_H_6_O_6_·C_6_H_8_N_6_·2H_2_O, crystallizes in a layer structure where each sheet is composed of anellated hydrogen-bonded rings of six distinct sizes: *R*_2_^2^(16), *R*_3_^3^(18), *R*_4_^4^(12), *R*_4_^4^(18), *R*_4_^4^(22) and *R*_4_^4^(25). The two largest rings, viz. *R*_4_^4^(22) and *R*_4_^4^(25), are associated with O—H⋯N bonds from the carboxyl groups to the triazole rings. The typical head-to-tail carbox­yl–carboxyl *R*_2_^2^(8) motif is not observed.

## Related literature

For related literature, see: Althoff *et al.* (2006 ▶); Dale & Elsegood (2004 ▶); Dale *et al.* (2004 ▶); Dorn *et al.* (2005 ▶, 2006 ▶); Du *et al.* (2005 ▶); Etter *et al.* (1990 ▶); Fan *et al.* (2005 ▶); Goldberg & Bernstein (2007 ▶); Janiak (2000 ▶); Shattock *et al.* (2005 ▶); Turner *et al.* (2008 ▶); Wang & Wang (2005 ▶); Wisser & Janiak (2007*a* ▶,*b* ▶).
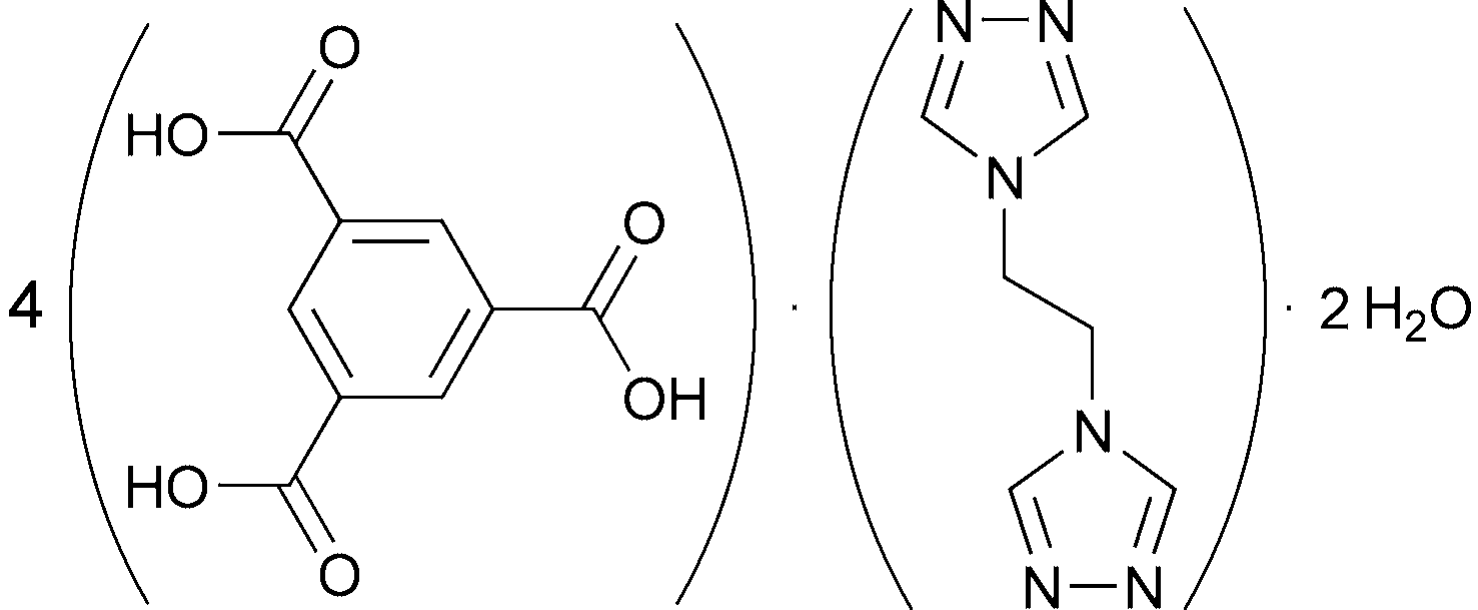


## Experimental

### 

#### Crystal data

4C_9_H_6_O_6_·C_6_H_8_N_6_·2H_2_O
*M* *_r_* = 1040.76
Triclinic, 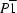

*a* = 9.7989 (1) Å
*b* = 10.7511 (2) Å
*c* = 12.6578 (2) Å
α = 108.801 (1)°
β = 98.737 (1)°
γ = 113.340 (1)°
*V* = 1097.44 (3) Å^3^
*Z* = 1
Mo *K*α radiation
μ = 0.13 mm^−1^
*T* = 203 (2) K
0.37 × 0.05 × 0.02 mm

#### Data collection

Bruker APEXII CCD area-detector diffractometer
Absorption correction: multi-scan (*SADABS*; Sheldrick, 1996 ▶) *T*_min_ = 0.952, *T*_max_ = 0.997
20984 measured reflections
4824 independent reflections
3452 reflections with *I* > 2σ(*I*)
*R*_int_ = 0.036

#### Refinement

*R*[*F* ^2^ > 2σ(*F* ^2^)] = 0.041
*wR*(*F* ^2^) = 0.105
*S* = 1.02
4824 reflections
359 parameters
H atoms treated by a mixture of independent and constrained refinement
Δρ_max_ = 0.25 e Å^−3^
Δρ_min_ = −0.24 e Å^−3^

### 

Data collection: *APEX2* (Bruker, 2006 ▶); cell refinement: *SAINT* (Bruker, 2006 ▶); data reduction: *SAINT*; program(s) used to solve structure: *SHELXS97* (Sheldrick, 2008 ▶); program(s) used to refine structure: *SHELXL97* (Sheldrick, 2008 ▶); molecular graphics: *DIAMOND* (Crystal Impact, 2006 ▶); software used to prepare material for publication: *publCIF* (Westrip, 2008 ▶).

## Supplementary Material

Crystal structure: contains datablocks I, global. DOI: 10.1107/S1600536808015808/kj2089sup1.cif

Structure factors: contains datablocks I. DOI: 10.1107/S1600536808015808/kj2089Isup2.hkl

Additional supplementary materials:  crystallographic information; 3D view; checkCIF report

## Figures and Tables

**Table 1:** Hydrogen-bond geometry (Å, °)

*D*—H⋯*A*	*D*—H	H⋯*A*	*D*⋯*A*	*D*—H⋯*A*
O2—H2⋯O11^i^	0.92 (2)	1.67 (2)	2.594 (2)	175 (2)
O4—H4⋯O8	0.88 (2)	1.75 (2)	2.626 (2)	174 (2)
O6—H6⋯O1^ii^	0.91 (2)	1.84 (2)	2.699 (2)	157 (2)
O7—H7⋯N1	0.96 (2)	1.75 (2)	2.703 (2)	172 (2)
O9—H9⋯O13	0.91 (3)	1.63 (3)	2.531 (2)	170 (2)
O12—H12⋯N2^iii^	0.90 (2)	1.76 (2)	2.644 (2)	167 (2)
O13—H13*A*⋯O10^iv^	0.91 (2)	1.81 (3)	2.711 (2)	171 (2)
O13—H13*B*⋯O5	0.85 (3)	1.92 (3)	2.751 (2)	167 (2)

Symmetry codes: (i) 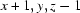; (ii) 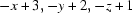; (iii) 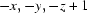; (iv) 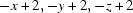.

**Fig. 1.**
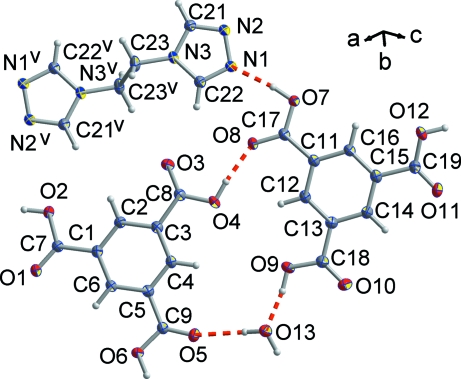
Fully labelled displacement ellipsoid diagram (at 50% probability) of the asymmetric unit. Symmetry code (v) 1-x, -y, -z.

**Fig. 2.**
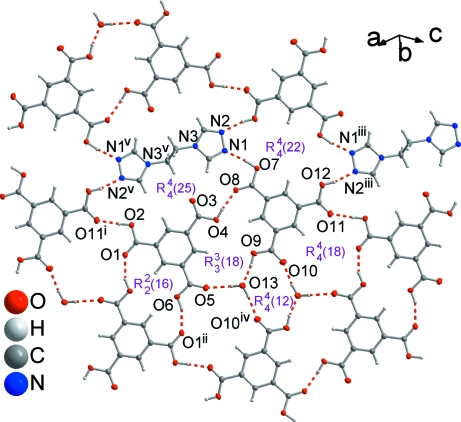
The hydrogen-bonded sheet in the structure of 2(C6H3-1,3,5-(COOH)3).0.5(C6H8N6).H2O with graph set pattern of hydrogen-bonded rings in violet. Hydrogen bond data is given in Table 1. Additional symmetry code (v) 1-x, -y, -z.

**Fig. 3.**
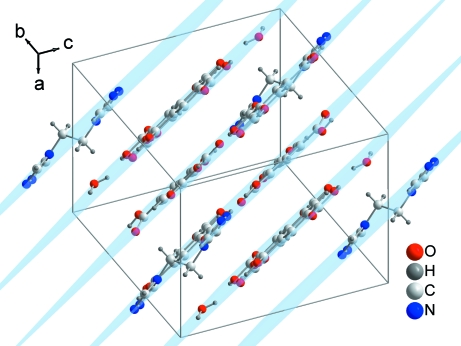
Packing of the hydrogen-bonded sheets parallel to the (-2,3,-2)-plane with a d-spacing (distance of neighboring sheets) of 3.155 Å.

## supplementary crystallographic information

### Comment

Hydrogen bonding within crystalline systems is of timely interest for the rational design of organized solids (Althoff *et al.*, 2006; Dorn *et al*., 2005; Dorn *et al.* 2006; Wisser & Janiak, 2007*a*,*b*). Co-crystallization of benzene-di, -tri- and -tetra-carboxylic acids, like trimesic acid or hemimellitic acid with solvent molecules or nitrogen bases is the focus of permanent and recent research activities (Dale & Elsegood, 2004; Dale *et al.*, 2004; Du *et al.*, 2005; Fan *et al.*, 2005; Goldberg & Bernstein, 2007; Shattock *et al.*, 2005; Turner *et al.*, 2008; Wang & Wang, 2005). Co-crystal structures of trimesic acid (benzene-1,3,5-tricarboxylic acid) have been reported with 2,5-bis(3- and 4-pyridyl)-1,3,4-oxadiazole (two-dimensional sheet, Du *et al.*, 2005), 3,6-bis(3'-pyridyl)-1,2,4,5-tetrazine (one-dimensional ribbon, Wang & Wang, 2005), 1,2-bis(4-pyridyl)ethane (two-dimensional 6,3- and 10,3-network with interpenetration, Shattock *et al.*, 2005), mono- and bis(methanol) (one-dimensional tape, Dale *et al.*, 2004), acetic acid (Goldberg & Bernstein, 2007) and dihydrate (three-dimensional network, Fan *et al.*, 2005).

The hydrogen-bonded sheet in the title compound contains several different motifs that engage all of the strong hydrogen bond donors and acceptors available (Fig. 1 and 2). The hydrogen bond distances in the sheet are spread over a narrow range, with D···A distances from 2.53 to 2.75 Å. The sheet is constructed of six distinct hydrogen-bonded rings of sizes *R*_2_^2^(16), *R*_3_^3^(18), *R*_4_^4^(12), *R*_4_^4^(18), *R*_4_^4^(22) and *R*_4_^4^(25), using Etter's graph set analysis (Etter *et al.*, 1990). The two largest rings *R*_4_^4^(22) and *R*_4_^4^(25) are associated with the O—H···N bonds from the carboxylic acid groups to the triazole rings. All N1 and N2 nitrogen atoms of the 1,2-bis(1,2,4-triazol-4-yl)ethane molecule act as hydrogen-bond acceptors. The smallest ring *R*_4_^4^(12) incorporates two water molecules and two carboxylic acid groups. The 18-membered *R*_3_^3^(18) and *R*_4_^4^(18) rings are constructed from one water molecule in combination with three and four carboxylic acid groups, respectively. These water and carboxylic acid containing motifs are different from those seen in the structure of the trimesic acid dihydrate (Fan *et al.*, 2005). Also, formation of the common *R*_2_^2^(8) head-to-tail carboxylic acid–acid graph-set motif is apparently prevented in the structure of the title compound by the water and the bis-triazole molecule. No relevant π–π or C—H···π interactions are found between molecules in adjacent sheets (Fig. 3) (Janiak, 2000).

### Experimental

A mixture of trimesic acid, H_3_btc (210 mg, 1.00 mmol), 1,2-bis(1,2,4-triazol-4-yl)ethane, btre (164 mg, 1.00 mmol) and water (15 ml) was stirred for 30 min at room temperature, transferred to a Teflon-lined stainless-steel autoclave and heated at 453 K for 3 d. Then the autoclave was cooled to room temperature at a rate of 2.8 K h^-1^. A colorless crystalline product was filtered off, washed with distilled water and dried in air (yield 135 mg, 52% based on H_3_btc). Elemental analysis C_21_H_18_N_3_O_13_ (520.38) calcd. C 48.47, H 3.49, N 8.07; found: C 47.84, H 3.49, N 8.02%.

### Refinement

Hydrogen atoms for aromatic CH and aliphatic CH_2_ were positioned geometrically (C—H = 0.94 Å for aromatic CH, C—H = 0.98 Å for CH_2_) and refined using a riding model. Protic hydrogen atoms of the carboxyl groups and of the water of crystallization were found and refined with *U*_iso_(H) = 1.5*U*_eq_(O).

### Crystal data

**Table:**

4C_9_H_6_O_6_·C_6_H_8_N_6_·2H_2_O	*Z* = 1
*M**_r_* = 1040.76	*F*_000_ = 538
Triclinic, *P*1	*D*_x_ = 1.575 Mg m^−^^3^
Hall symbol: -P 1	Mo *K*α radiation λ = 0.71073 Å
*a* = 9.7989 (1) Å	Cell parameters from 5028 reflections
*b* = 10.7511 (2) Å	θ = 2.2–31.5º
*c* = 12.6578 (2) Å	µ = 0.13 mm^−^^1^
α = 108.801 (1)º	*T* = 203 (2) K
β = 98.737 (1)º	Needle, colourless
γ = 113.340 (1)º	0.37 × 0.05 × 0.02 mm
*V* = 1097.44 (3) Å^3^	

### Data collection

**Table:**

Bruker APEXII CCD area-detector diffractometer	4824 independent reflections
Radiation source: fine-focus sealed tube	3452 reflections with *I* > 2σ(*I*)
Monochromator: graphite	*R*_int_ = 0.036
*T* = 203(2) K	θ_max_ = 27.1º
ω scans	θ_min_ = 1.8º
Absorption correction: multi-scan(SADABS; Sheldrick, 1996)	*h* = −12→12
*T*_min_ = 0.952, *T*_max_ = 0.997	*k* = −13→13
20984 measured reflections	*l* = −16→16

### Refinement

**Table:**

Refinement on *F*^2^	Secondary atom site location: difference Fourier map
Least-squares matrix: full	Hydrogen site location: inferred from neighbouring sites
*R*[*F*^2^ > 2σ(*F*^2^)] = 0.041	H atoms treated by a mixture of independent and constrained refinement
*wR*(*F*^2^) = 0.105	*w* = 1/[σ^2^(*F*_o_^2^) + (0.0562*P*)^2^ + 0.0985*P*] where *P* = (*F*_o_^2^ + 2*F*_c_^2^)/3
*S* = 1.02	(Δ/σ)_max_ < 0.001
4824 reflections	Δρ_max_ = 0.25 e Å^−^^3^
359 parameters	Δρ_min_ = −0.24 e Å^−^^3^
Primary atom site location: structure-invariant direct methods	Extinction correction: none

### Special details

**Table:**

Experimental. IR (KBr) 3512*m* (νCOO-H), 3427*m* (νCOO-H), 3122*m*, 1886*m*, 1704 s, (ν*_asym_*CO_2_), 1539*m* (ν*_asym_*CO_2_),1452 s (ν*_sym_*CO_2_), 1356w (ν*_sym_*CO_2_), 1320w (δOH···O),1285*m*, 1225*m*, 1190*m*, 1071*m*, 1020*m*, 986*m*, 936*m* (γOH···O), 905*m*, 870w, 844w, 814w, 745 s, 683 s, 666 s, 936*m*, 605w, 570w, 510*m*, 448*m* cm^-1^.Thermogravimetric analysis (simultaneous thermoanalysis apparatus STA 409 C from Netzsch under nitrogen with a heating rate of 10 K min^-1^ in the range of 323 to 920 K): A sample of the compound shows the first weight loss in the temperature range 450–490 K which corresponds to the removal of the water molecule (obs. 3.67, calcd. 3.45%). From 550 to 610 K a less well resolved weight loss of about 17% occurs which is assigned to the half btre molecule (calcd. 15.8%). A third weight loss in the range 610–650 K of around 40% is assigned to the removal of one H~3~btc molecule (calcd. 40.3%). A weight loss continues to 920 K where 18.6% of the original mass is retained.
Geometry. All e.s.d.'s (except the e.s.d. in the dihedral angle between two l.s. planes) are estimated using the full covariance matrix. The cell e.s.d.'s are taken into account individually in the estimation of e.s.d.'s in distances, angles and torsion angles; correlations between e.s.d.'s in cell parameters are only used when they are defined by crystal symmetry. An approximate (isotropic) treatment of cell e.s.d.'s is used for estimating e.s.d.'s involving l.s. planes.
Refinement. Refinement of *F*^2^ against ALL reflections. The weighted *R*-factor *wR* and goodness of fit *S* are based on *F*^2^, conventional *R*-factors *R* are based on *F*, with *F* set to zero for negative *F*^2^. The threshold expression of *F*^2^ > σ(*F*^2^) is used only for calculating *R*-factors(gt) *etc*. and is not relevant to the choice of reflections for refinement. *R*-factors based on *F*^2^ are statistically about twice as large as those based on *F*, and *R*- factors based on ALL data will be even larger.

### Fractional atomic coordinates and isotropic or equivalent isotropic displacement parameters (Å^2^)

**Table:**

	*x*	*y*	*z*	*U*_iso_*/*U*_eq_	
O1	1.35077 (13)	0.71622 (12)	0.20868 (9)	0.0281 (3)	
O2	1.12433 (15)	0.50380 (13)	0.11195 (10)	0.0342 (3)	
H2	1.169 (2)	0.490 (2)	0.0526 (19)	0.051*	
O3	0.72628 (14)	0.32810 (13)	0.28747 (10)	0.0357 (3)	
O4	0.75183 (14)	0.47603 (13)	0.46864 (10)	0.0295 (3)	
H4	0.658 (3)	0.402 (2)	0.4509 (17)	0.044*	
O5	1.23563 (17)	0.97506 (14)	0.69889 (11)	0.0479 (4)	
O6	1.41605 (14)	1.03213 (13)	0.61107 (11)	0.0335 (3)	
H6	1.473 (3)	1.117 (2)	0.6791 (19)	0.050*	
C1	1.15857 (19)	0.65177 (17)	0.30595 (13)	0.0226 (3)	
C2	1.01588 (19)	0.54260 (17)	0.29865 (13)	0.0241 (4)	
H2A	0.9603	0.4520	0.2306	0.031 (5)*	
C3	0.95434 (19)	0.56593 (17)	0.39110 (13)	0.0224 (3)	
C4	1.03774 (18)	0.69911 (17)	0.49313 (13)	0.0230 (3)	
H4A	0.9963	0.7159	0.5555	0.028*	
C5	1.18299 (19)	0.80705 (17)	0.50200 (13)	0.0228 (3)	
C6	1.24244 (19)	0.78375 (17)	0.40836 (13)	0.0230 (3)	
H6A	1.3397	0.8576	0.4144	0.028*	
C7	1.22269 (19)	0.62928 (17)	0.20573 (13)	0.0235 (3)	
C8	0.79976 (19)	0.44429 (17)	0.37606 (14)	0.0237 (3)	
C9	1.2777 (2)	0.94488 (18)	0.61364 (14)	0.0260 (4)	
O7	0.23309 (14)	0.11931 (13)	0.42638 (10)	0.0327 (3)	
H7	0.230 (2)	0.064 (2)	0.349 (2)	0.049*	
O8	0.47138 (14)	0.26410 (13)	0.43007 (10)	0.0369 (3)	
O9	0.82100 (16)	0.72343 (14)	0.77430 (11)	0.0405 (3)	
H9	0.916 (3)	0.808 (3)	0.814 (2)	0.061*	
O10	0.75891 (16)	0.79239 (14)	0.93837 (11)	0.0487 (4)	
O11	0.23548 (16)	0.46243 (14)	0.93776 (11)	0.0443 (4)	
O12	0.10133 (15)	0.22723 (13)	0.79990 (11)	0.0360 (3)	
H12	0.029 (3)	0.214 (2)	0.8372 (19)	0.054*	
C11	0.40237 (18)	0.33372 (17)	0.60392 (13)	0.0226 (3)	
C12	0.54215 (19)	0.46733 (17)	0.66014 (14)	0.0248 (4)	
H12A	0.6137	0.4932	0.6193	0.030*	
C13	0.57695 (19)	0.56305 (17)	0.77641 (14)	0.0252 (4)	
C14	0.47026 (19)	0.52582 (17)	0.83609 (14)	0.0259 (4)	
H14A	0.4929	0.5908	0.9144	0.031*	
C15	0.33011 (19)	0.39277 (17)	0.78027 (14)	0.0242 (4)	
C16	0.29563 (19)	0.29562 (17)	0.66414 (13)	0.0226 (3)	
H16A	0.2012	0.2052	0.6267	0.027*	
C17	0.37241 (19)	0.23634 (17)	0.47946 (14)	0.0255 (4)	
C18	0.7287 (2)	0.70561 (18)	0.83851 (15)	0.0295 (4)	
C19	0.2168 (2)	0.36255 (19)	0.84716 (14)	0.0276 (4)	
O13	1.09496 (16)	0.94370 (15)	0.86765 (12)	0.0442 (4)	
H13A	1.134 (3)	1.031 (3)	0.932 (2)	0.066*	
H13B	1.129 (3)	0.959 (3)	0.813 (2)	0.066*	
C21	0.2033 (2)	−0.17823 (19)	0.03864 (14)	0.0307 (4)	
H21A	0.1519	−0.2613	−0.0349	0.037*	
C22	0.3793 (2)	0.02501 (18)	0.18472 (14)	0.0277 (4)	
H22A	0.4747	0.1115	0.2332	0.033*	
C23	0.4714 (2)	−0.0680 (2)	0.01332 (16)	0.0338 (4)	
H23A	0.4241	−0.1585	−0.0605	0.041*	
H23B	0.5608	−0.0657	0.0623	0.041*	
N1	0.25099 (16)	−0.01876 (15)	0.21283 (12)	0.0281 (3)	
N2	0.13810 (16)	−0.14927 (15)	0.11899 (12)	0.0293 (3)	
N3	0.35506 (16)	−0.07258 (15)	0.07521 (11)	0.0272 (3)	

### Atomic displacement parameters (Å^2^)

**Table:**

	*U*^11^	*U*^22^	*U*^33^	*U*^12^	*U*^13^	*U*^23^
O1	0.0239 (6)	0.0268 (6)	0.0238 (6)	0.0049 (5)	0.0126 (5)	0.0063 (5)
O2	0.0292 (7)	0.0311 (7)	0.0215 (6)	0.0013 (6)	0.0140 (5)	0.0010 (5)
O3	0.0264 (7)	0.0300 (7)	0.0276 (7)	−0.0011 (6)	0.0100 (5)	0.0032 (6)
O4	0.0195 (6)	0.0265 (6)	0.0300 (6)	0.0014 (5)	0.0130 (5)	0.0073 (5)
O5	0.0467 (9)	0.0343 (7)	0.0338 (7)	0.0005 (6)	0.0268 (7)	−0.0017 (6)
O6	0.0236 (7)	0.0270 (6)	0.0248 (6)	−0.0018 (5)	0.0099 (5)	−0.0015 (5)
C1	0.0207 (8)	0.0239 (8)	0.0197 (8)	0.0080 (7)	0.0084 (7)	0.0075 (7)
C2	0.0208 (9)	0.0237 (8)	0.0187 (8)	0.0054 (7)	0.0065 (7)	0.0049 (7)
C3	0.0187 (8)	0.0223 (8)	0.0225 (8)	0.0070 (7)	0.0074 (7)	0.0081 (7)
C4	0.0209 (8)	0.0235 (8)	0.0221 (8)	0.0079 (7)	0.0111 (7)	0.0082 (7)
C5	0.0207 (9)	0.0219 (8)	0.0228 (8)	0.0079 (7)	0.0091 (7)	0.0078 (7)
C6	0.0178 (8)	0.0226 (8)	0.0229 (8)	0.0050 (7)	0.0093 (7)	0.0076 (7)
C7	0.0211 (9)	0.0219 (8)	0.0209 (8)	0.0058 (7)	0.0084 (7)	0.0065 (7)
C8	0.0191 (8)	0.0245 (8)	0.0240 (8)	0.0069 (7)	0.0083 (7)	0.0100 (7)
C9	0.0248 (9)	0.0218 (8)	0.0250 (8)	0.0062 (7)	0.0131 (7)	0.0065 (7)
O7	0.0237 (7)	0.0300 (6)	0.0214 (6)	−0.0013 (5)	0.0110 (5)	0.0004 (5)
O8	0.0273 (7)	0.0332 (7)	0.0261 (6)	−0.0017 (6)	0.0167 (6)	0.0013 (5)
O9	0.0282 (7)	0.0291 (7)	0.0359 (7)	−0.0047 (6)	0.0161 (6)	0.0014 (6)
O10	0.0416 (8)	0.0347 (7)	0.0305 (7)	−0.0044 (6)	0.0152 (6)	−0.0049 (6)
O11	0.0399 (8)	0.0402 (8)	0.0307 (7)	0.0046 (6)	0.0237 (6)	0.0018 (6)
O12	0.0269 (7)	0.0324 (7)	0.0349 (7)	0.0026 (6)	0.0202 (6)	0.0079 (6)
C11	0.0199 (9)	0.0220 (8)	0.0225 (8)	0.0073 (7)	0.0090 (7)	0.0081 (7)
C12	0.0223 (9)	0.0240 (8)	0.0255 (8)	0.0077 (7)	0.0123 (7)	0.0095 (7)
C13	0.0234 (9)	0.0218 (8)	0.0242 (8)	0.0072 (7)	0.0094 (7)	0.0063 (7)
C14	0.0243 (9)	0.0239 (8)	0.0220 (8)	0.0075 (7)	0.0101 (7)	0.0051 (7)
C15	0.0223 (9)	0.0251 (8)	0.0245 (8)	0.0096 (7)	0.0109 (7)	0.0101 (7)
C16	0.0197 (8)	0.0208 (8)	0.0223 (8)	0.0060 (7)	0.0083 (7)	0.0072 (7)
C17	0.0219 (9)	0.0237 (8)	0.0240 (8)	0.0051 (7)	0.0104 (7)	0.0082 (7)
C18	0.0273 (10)	0.0236 (9)	0.0274 (9)	0.0054 (8)	0.0115 (8)	0.0060 (7)
C19	0.0242 (9)	0.0311 (9)	0.0227 (8)	0.0092 (8)	0.0109 (7)	0.0094 (7)
O13	0.0374 (8)	0.0341 (7)	0.0289 (7)	−0.0054 (6)	0.0157 (6)	0.0019 (6)
C21	0.0262 (9)	0.0272 (9)	0.0209 (8)	0.0011 (7)	0.0097 (7)	0.0032 (7)
C22	0.0236 (9)	0.0258 (8)	0.0229 (8)	0.0040 (7)	0.0103 (7)	0.0062 (7)
C23	0.0336 (10)	0.0365 (10)	0.0329 (9)	0.0138 (8)	0.0233 (8)	0.0144 (8)
N1	0.0233 (8)	0.0249 (7)	0.0228 (7)	0.0023 (6)	0.0103 (6)	0.0049 (6)
N2	0.0223 (8)	0.0280 (7)	0.0234 (7)	0.0022 (6)	0.0105 (6)	0.0053 (6)
N3	0.0239 (8)	0.0268 (7)	0.0235 (7)	0.0055 (6)	0.0132 (6)	0.0079 (6)

### Geometric parameters (Å, °)

**Table:**

O1—C7	1.2182 (19)	O12—C19	1.298 (2)
O2—C7	1.3221 (19)	O12—H12	0.90 (2)
O2—H2	0.92 (2)	C11—C12	1.390 (2)
O3—C8	1.2147 (19)	C11—C16	1.395 (2)
O4—C8	1.3217 (19)	C11—C17	1.489 (2)
O4—H4	0.88 (2)	C12—C13	1.391 (2)
O5—C9	1.2065 (19)	C12—H12A	0.9400
O6—C9	1.3204 (19)	C13—C14	1.388 (2)
O6—H6	0.91 (2)	C13—C18	1.497 (2)
C1—C2	1.390 (2)	C14—C15	1.389 (2)
C1—C6	1.391 (2)	C14—H14A	0.9400
C1—C7	1.492 (2)	C15—C16	1.395 (2)
C2—C3	1.393 (2)	C15—C19	1.494 (2)
C2—H2A	0.9400	C16—H16A	0.9400
C3—C4	1.395 (2)	O13—H13A	0.91 (2)
C3—C8	1.491 (2)	O13—H13B	0.85 (3)
C4—C5	1.394 (2)	C21—N2	1.300 (2)
C4—H4A	0.9400	C21—N3	1.353 (2)
C5—C6	1.393 (2)	C21—H21A	0.9400
C5—C9	1.489 (2)	C22—N1	1.306 (2)
C6—H6A	0.9400	C22—N3	1.358 (2)
O7—C17	1.3090 (19)	C22—H22A	0.9400
O7—H7	0.96 (2)	C23—N3	1.472 (2)
O8—C17	1.2230 (18)	C23—C23^i^	1.513 (3)
O9—C18	1.304 (2)	C23—H23A	0.9800
O9—H9	0.91 (3)	C23—H23B	0.9800
O10—C18	1.210 (2)	N1—N2	1.3784 (18)
O11—C19	1.2201 (19)		
			
C7—O2—H2	110.6 (13)	C14—C13—C12	119.73 (15)
C8—O4—H4	107.2 (13)	C14—C13—C18	119.44 (14)
C9—O6—H6	112.6 (13)	C12—C13—C18	120.83 (14)
C2—C1—C6	119.32 (14)	C13—C14—C15	120.10 (15)
C2—C1—C7	120.95 (14)	C13—C14—H14A	120.0
C6—C1—C7	119.73 (14)	C15—C14—H14A	120.0
C1—C2—C3	120.72 (14)	C14—C15—C16	120.34 (14)
C1—C2—H2A	119.6	C14—C15—C19	117.53 (14)
C3—C2—H2A	119.6	C16—C15—C19	122.06 (15)
C2—C3—C4	119.85 (14)	C15—C16—C11	119.51 (15)
C2—C3—C8	117.56 (14)	C15—C16—H16A	120.2
C4—C3—C8	122.59 (14)	C11—C16—H16A	120.2
C5—C4—C3	119.53 (14)	O8—C17—O7	122.24 (14)
C5—C4—H4A	120.2	O8—C17—C11	122.17 (15)
C3—C4—H4A	120.2	O7—C17—C11	115.59 (13)
C6—C5—C4	120.21 (14)	O10—C18—O9	125.16 (16)
C6—C5—C9	119.87 (14)	O10—C18—C13	121.74 (15)
C4—C5—C9	119.88 (13)	O9—C18—C13	113.10 (14)
C1—C6—C5	120.34 (14)	O11—C19—O12	125.08 (15)
C1—C6—H6A	119.8	O11—C19—C15	120.02 (15)
C5—C6—H6A	119.8	O12—C19—C15	114.90 (14)
O1—C7—O2	123.19 (14)	H13A—O13—H13B	110 (2)
O1—C7—C1	124.36 (15)	N2—C21—N3	110.82 (14)
O2—C7—C1	112.45 (13)	N2—C21—H21A	124.6
O3—C8—O4	123.84 (15)	N3—C21—H21A	124.6
O3—C8—C3	123.02 (14)	N1—C22—N3	110.10 (15)
O4—C8—C3	113.14 (14)	N1—C22—H22A	124.9
O5—C9—O6	122.58 (16)	N3—C22—H22A	124.9
O5—C9—C5	124.55 (15)	N3—C23—C23^i^	111.15 (18)
O6—C9—C5	112.86 (13)	N3—C23—H23A	109.4
C17—O7—H7	107.8 (13)	C23^i^—C23—H23A	109.4
C18—O9—H9	112.4 (14)	N3—C23—H23B	109.4
C19—O12—H12	113.6 (14)	C23^i^—C23—H23B	109.4
C12—C11—C16	119.86 (14)	H23A—C23—H23B	108.0
C12—C11—C17	117.73 (14)	C22—N1—N2	107.37 (13)
C16—C11—C17	122.41 (14)	C21—N2—N1	106.94 (13)
C11—C12—C13	120.45 (14)	C21—N3—C22	104.77 (13)
C11—C12—H12A	119.8	C21—N3—C23	127.89 (14)
C13—C12—H12A	119.8	C22—N3—C23	127.25 (14)

Symmetry codes: (i) −*x*+1, −*y*, −*z*.

### Hydrogen-bond geometry (Å, °)

**Table:**

*D*—H···*A*	*D*—H	H···*A*	*D*···*A*	*D*—H···*A*
O2—H2···O11^ii^	0.92 (2)	1.67 (2)	2.594 (2)	175 (2)
O4—H4···O8	0.88 (2)	1.75 (2)	2.626 (2)	174 (2)
O6—H6···O1^iii^	0.91 (2)	1.84 (2)	2.699 (2)	157 (2)
O7—H7···N1	0.96 (2)	1.75 (2)	2.703 (2)	172 (2)
O9—H9···O13	0.91 (3)	1.63 (3)	2.531 (2)	170 (2)
O12—H12···N2^iv^	0.90 (2)	1.76 (2)	2.644 (2)	167 (2)
O13—H13A···O10^v^	0.91 (2)	1.81 (3)	2.711 (2)	171 (2)
O13—H13B···O5	0.85 (3)	1.92 (3)	2.751 (2)	167 (2)

Symmetry codes: (ii) *x*+1, *y*, *z*−1; (iii) −*x*+3, −*y*+2, −*z*+1; (iv) −*x*, −*y*, −*z*+1; (v) −*x*+2, −*y*+2, −*z*+2.
